# Effects of Tiaopi Xiezhuo decoction on constipation and gut dysbiosis in patients with peritoneal dialysis

**DOI:** 10.1080/13880209.2023.2193595

**Published:** 2023-03-30

**Authors:** Yu Peng, Yuting Zeng, Tingting Zheng, Xiaoning Xie, Jianfeng Wu, Lizhe Fu, Fuhua Lu, La Zhang, Yang Chen, Xusheng Liu, Lei Wang

**Affiliations:** a Department of Nephrology, Second Affiliated Hospital of Guangzhou University of Traditional Chinese Medicine, Guangzhou University of Chinese Medicine, Guangzhou, P.R. China; b School of Pharmaceutical Sciences, Guangzhou University of Chinese Medicine, Guangzhou, P.R. China; c Guangdong Provincial Key Laboratory of Clinical Research on Traditional Chinese Medicine Syndrome, Second Affiliated Hospital of Guangzhou University of Traditional Chinese Medicine, Guangzhou University of Chinese Medicine, Guangzhou, P.R. China

**Keywords:** Clinical study, end-stage kidney disease, peritonitis, gut microbiota, probiotic bacteria

## Abstract

**Context:**

A Chinese herbal formula, Tiaopi Xiezhuo decoction (TXD), is developed from a classical Chinese prescription Sanhuang Xiexin decoction.

**Objective:**

To investigate the regulatory effect of TXD on gut dysbiosis, as a treatment of constipation in patients with peritoneal dialysis (PD).

**Materials and methods:**

The chemical content of TXD was assessed by high-performance liquid chromatography. A total of 29 PD patients were enrolled and treated with TXD orally (3 g crude drug/each/twice/day) for 3 months. Blood and faecal samples were collected at the beginning and end, to determine the changes in biochemical characteristics and gut microbial composition. The stool conditions were asked to be scored. Additional 30 healthy individuals were recruited as a control for the analysis of gut microbiota.

**Results:**

Although having no significant effects on serum biochemical characteristics, 3-month TXD intervention improved constipation in PD patients: decreased 80% abdominal distention (*p* < 0.01), increased 2.6-fold sloppy stools (*p* < 0.05) and eliminated hard stool completely (*p* < 0.01). The analysis of gut microbiota showed that, compared to the healthy group, the microbial richness was reduced in PD patients. After a 3-month TXD treatment, this reduced richness was raised, and *Paraprevotella clara*, *Lachnospiraceae bacterium* 2-146FA, *Phascolarctobaterium succinatutens*, *Lachnospiraceae bacterium* 2-1-58FAA, *Fusobacterium mortiferum,* and *Prevotella copri* were accumulated in the intestinal flora. Furthermore, the bacterial species enriched by TXD correlated with the improvement of constipation.

**Discussion and conclusions:**

TXD treatment may improve constipation by modulating gut dysbiosis in PD patients. These findings provide data to support the further application of TXD in the adjuvant treatment of PD.

## Introduction

Peritoneal dialysis (PD) is one of the three main alternative therapies for end-stage kidney disease (ESKD). PD uses the patient’s peritoneal tissue to exchange water, creatinine, urea, electrolytes, glucose, and other small and medium-sized toxins between the capillaries on either side of the peritoneum and dialysate (Teitelbaum 2021). However, certain complications discount the therapeutic effect of PD. For example, constipation is highly prevalent in these patients and affects their quality of life (Longstreth et al. 2006; Cano et al. 2007; Kosmadakis et al. 2019). Constipation is associated with an increased risk of bacterial translocation and eventual enteric peritonitis. Peritonitis results in considerable morbidity, mortality, and health care costs, as well as limitations in PD modality (Cho and Johnson 2014). Thus, there is an urgent need to develop strategies that reduce constipation and prolong the treatment time of PD.

Tiaopi Xiezhuo decoction (TXD), a Chinese herbal formula, is developed from the historical Sanhuang Xiexin decoction which was first described in the *Synopsis of Golden Chamber* by Zhongjing Zhang of the Eastern Han Dynasty. To improve gastrointestinal symptoms and limit liquid intake in patients with dialysis, the recipe of TXD was modified according to medical records and finally consists of *Rheum palmatum* L. (Polygonaceae), *Coptis chinensis* Franch (Ranunculaceae), and *Zingiber officinale* Roscoe (Zingiberaceae) at a ratio of 1:1:1. Among them, *R. palmatum* is a traditional herbal medicine for the treatment of constipation. Our clinical data showed that TXD significantly improved gastrointestinal symptoms in PD patients (Liang et al. 2020) but the underlying mechanism is still unknown. It is noticed that all three herbs in TXD are reported to have antibacterial properties (Park et al. 2008; Wong et al. 2010; Feng et al. 2011). Recently, *R. palmatum* and *C. chinensis* have been found to modulate gut microbiota in rodent studies (Ji et al. 2020; Lyu et al. 2021). Therefore, we hypothesized that TXD may improve constipation by modulating gut dysbiosis in patients with PD. In response, we designed this study, where we recruited 29 PD patients and treated them with a 3-month TXD intervention, to estimate the changes in constipation situation and gut microbial structures at the baseline and post-intervention. The faecal samples of additional 30 healthy individuals were used as a control for the analysis of gut microbiota.

## Materials and methods

### Preparation and fingerprint analysis of Tiaopi Xiezhuo decoction

Tiaopi Xiezhuo decoction (TXD) is made by commercial granules of *R. palmatum* (1 g/bag), *C. chinensis* (0.5 g/bag), and *Z. officinale* (0.5 g/bag) at one bag of each for a time, which were produced by Jiangyin Tianjiang Pharmaceutical Co., Ltd. According to the manufacturer’s protocols, the roots of each herb have been decocted, filtered, concentrated and dried, and made into granules, respectively; each bag of granules is equivalent to 3 g of a crude drug; qualities of granules, such as appearance, determination of content, size of granule, solubility, hygroscopicity, heavy metals, toxic elements, pesticide residues, and microbial limit, were controlled rigorously according to the 2015 Chinese pharmacopoeia.

The chemical content of TXD solution was assessed by high-performance liquid chromatography (HPLC) using a COSMOSIL 5C18-MS-II column (4.6 × 250 mm, 5 µm, Nacalai Tesque, Inc., Kyoto, Japan) on an Agilent 1290 Infinity II LC system (Agilent, Cheadle, UK). The granules of TXD were dissolved in 50 mL of deionized water. After being filtered through a 0.45 µm membrane, 5 μL of TXD solution was carefully transferred to sample vials for HPLC analysis. The mobile phase consisted of acetonitrile (ThermoFisher Scientific, Shanghai, China) as solvent A and 0.2% formic acid (Merck, Shanghai, China) in water as solvent B. Then the elution was performed in mobile phase gradient (A:B) of 2:98, 15:85, 30:70 and 98:2 at a rate of 1 mL/min with UV detection at 280 nm. Standard epiberberine or berberine (Sichuan Victory Biological Technology Co., Ltd., Chengdu, China) were prepared in methanol at a concentration of 1 mg/mL and eluted in the mobile phase.

### Study design and subjects

**Figure 1.**
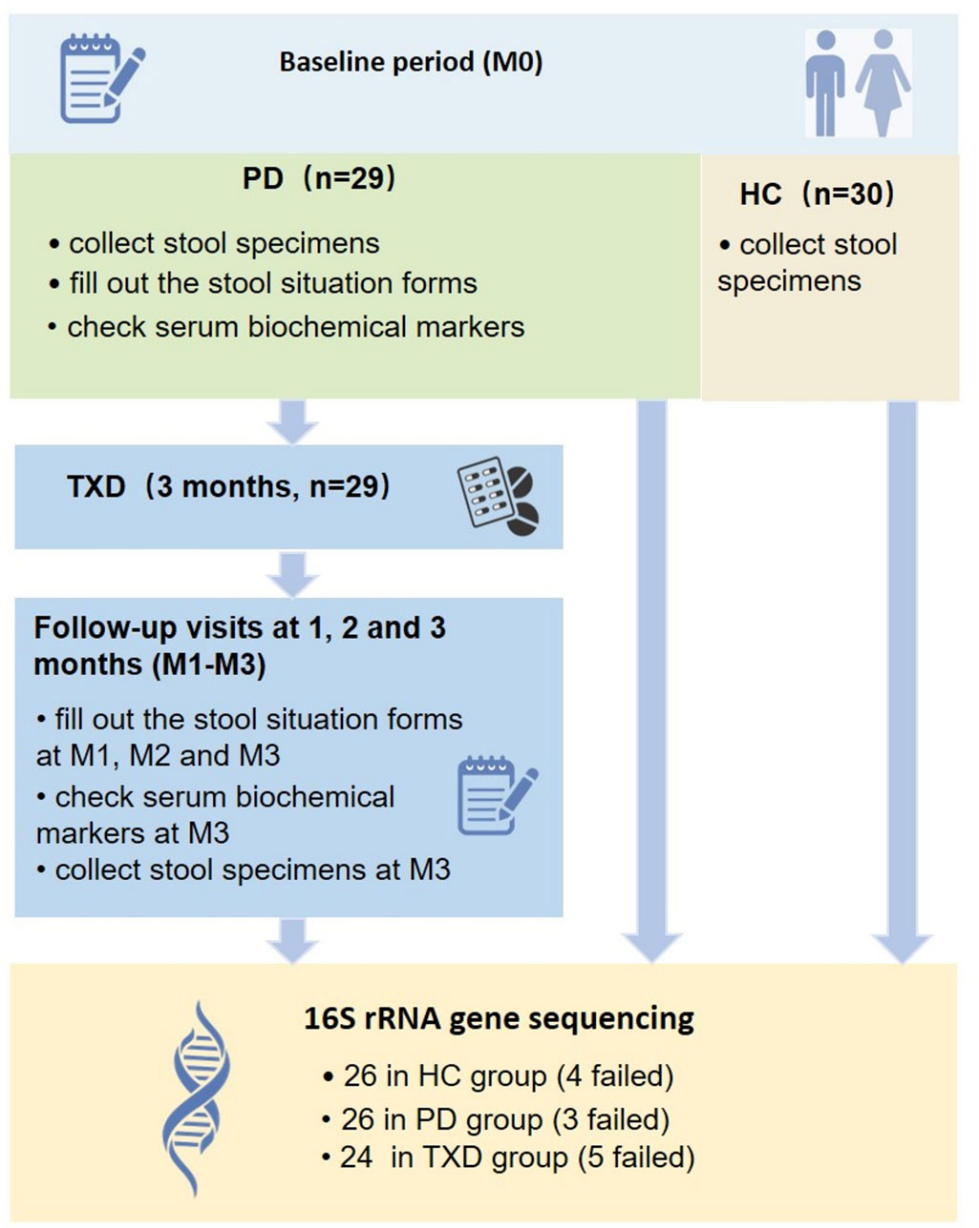
Clinical trial flow diagram.

We recruited 29 PD patients and 30 healthy individuals for this study. Among them, 29 randomized PD patients aged 18–80 years old, who had received stable PD for longer than 2 months, were enrolled on the peritoneal dialysis clinic of Guangdong Provincial Hospital of Traditional Chinese Medicine between December 2017 to December 2018; 30 healthy individuals were recruited from the families of PD patients, 18–80 years old, who have no chronic kidney diseases, cardiovascular diseases or cancers. We excluded subjects who showed infection within 1 month, had diarrhoea within 2 weeks and used hormones, antibiotics, or intestinal flora-regulatory drugs in the previous 3 months. We also excluded those with inflammatory bowel disease, intestinal malignancies, and tuberculosis. All the participants completed the ‘Information Collection Form’ upon enrollment in the study, stated that they were willing to cooperate with this research, and signed an informed consent form. The protocol was approved by the ethics committee of the Guangdong Provincial Hospital of Traditional Chinese Medicine (B2017-052.2-02), and the project has been registered at the China Clinical Trial Registration Center (ChiCTR-INR-17013624). The trial was performed in accordance with Good Clinical Practice guidelines. The clinical trial flow diagram is shown in Figure 1.

As shown in Figure 1, the stool specimen and serum sample of 29 PD (PD group) were collected at the baseline (M0). Subsequently, all 29 PD patients were given 3-month TXD intervention (orally, twice a day, TXD group), and asked to attend follow-up visits at the end of one, two, and three months (termed as M1, M2 and M3). At each visit, every patient was asked to fill out the forms about stool conditions. The stool specimens and serum samples were collected only after 3-month TXD treatment (M3). The stool specimens of the healthy group (HC group) were obtained as a control for the analysis of gut microbiota.

### Evaluation of constipation in PD patients

At the enrollment and follow-up visits (M0-M3), all 29 PD patients were asked to fill out forms ranking their stool experience, characterized as abdominal distention, sloppy stool, and hard stool, and provide a 0–4 score indicating light to heavy (Svedlund et al. 1988). The average scores were then calculated.

### Stool specimen collection

Between 10–20 g of stool specimens were collected for the analysis of microbiota from all 29 PD patients before and after the 3-month TXD intervention. All faecal specimens were stored in a refrigerator at −80 °C until assay. During the development of the following microbiota profiles, three specimens in the PD group and five in the TXD group failed to be sequenced. Additionally, 30 stool specimens were obtained from healthy individuals, but four were not successfully sequenced.

### Analysis of gut microbiota

All stool samples were sent to BGI Genomics Co., Ltd. for structural analysis of gut microbiota. Fecal bacterial DNA was extracted, and 16S rRNA gene sequence libraries were generated using the V3-V4 primer region on the Illumina MiSeq platform (Illumina, San Diego, CA, USA). The operational taxonomic units and α- or β-diversity indices were calculated based on the OTUs data using QIIME (version 1.8.0). Principal coordinates analysis (PCoA) was used to compare the community composition of gut bacteria between groups. Linear discriminant analysis Effect Size (LEfSe) was used to identify the specific species with the most significant differences. The HUMAnN2 method was used to directly compare metagenomics to the metabolic pathways of response genes. Metabolic pathways were mainly interpreted by referring to the MetaCYC database.

### Statistical analysis

Statistical analyses were performed using SPSS 18.0 statistical software (SPSS Inc., Chicago, USA). Data are presented as mean value ± standard error for normally distributed variables, or as medians for non-normally distributed variables. Spearman rank correlation was determined, using GraphPad Prism software (GraphPad Software, San Diego, USA), to analyze the relationship between the enriched species and clinical data. Statistical significance was set at *p* < 0.05.

## Results

### Fingerprinting analysis of TXD

**Figure 2.**
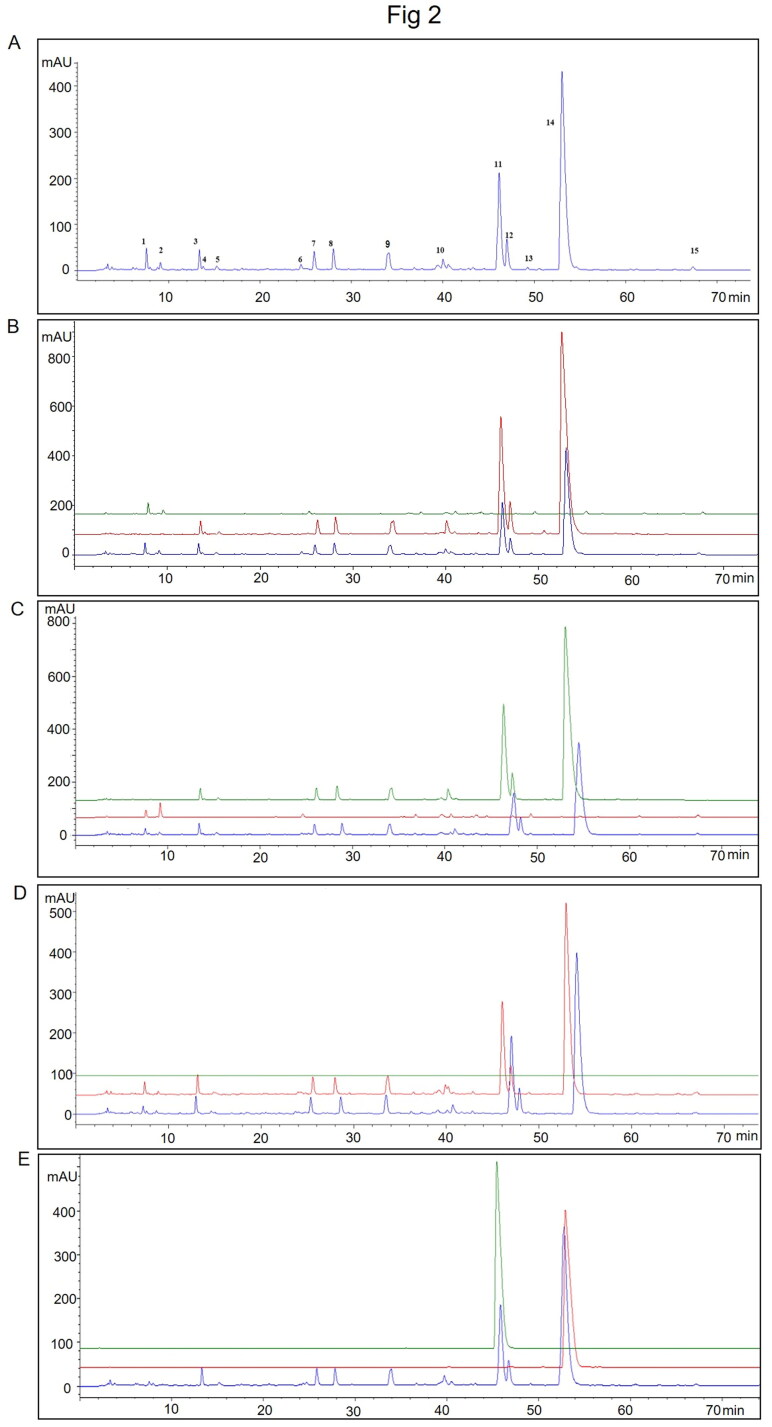
The ultra-high performance liquid chromatography fingerprints of TXD. (A) representative HPLC chromatograms of TXD. (B) chromatogram overlap of *R. palmatum* granules (green), TXD without *R. palmatum* granules (red) and TXD (blue). (C) chromatogram overlap of *C. chinensis* granules (green), TXD without *C. chinensis* granules (red) and TXD (blue). (D) chromatogram overlap of *Z. officinale* granules (green), TXD without *Z. officinale* granules (red) and TXD (blue). (E) chromatogram overlap of epiberberine (green), berberine (red) and TXD (blue).

Representative HPLC chromatograms of TXD solution are shown in Figure 2(A). Under our condition, 15 peaks are appearing in the solution. Among them, Peak 1, 2, 6, 10, 13, and 15 are from *R. palmatum* (Figure 2(B)); Peak 3, 4, 5, 7, 8, 9, 10, 11, 12, and 14 are from *C. chinensis* (Figure 2(C)). Under the same condition, we didn’t detect peaks from *Z. officinale* (Figure 2(D)). Additionally, compared to the standard Peaks of epiberberine and berberine, Peak 11 is epiberberine and Peak 14 is berberine (Figure 2(E)).

### Effects of TXD on clinical characteristics and constipation of PD patients

To estimate the effects of TXD on the clinical characteristics of PD patients, 22 biochemical markers were determined in the serum of PD patients before and after 3-month TXD intervention. As shown in Table 1, no significant changes were observed between the baseline and post-intervention.

**Table 1.** Clinical characteristics of PD patients before and after the TXD intervention.

	PD (*n* = 29)	TXD (*n* = 29)	*p* Value
WBC	6.68 ± 0.34	6.33 ± 0.33	*p* > 0.05
RBC	3.83 ± 0.16	3.71 ± 0.60	*p* > 0.05
HB	109.14 ± 3.34	106.10 ± 3.61	*p* > 0.05
Scr	1016.07 ± 45.31	1089.14 ± 37.08	*p* > 0.05
BUN	23.33 ± 1.07	23.77 ± 0.79	*p* > 0.05
UA	419.86 ± 13.08	423.89 ± 0.83	*p* > 0.05
GLU	5.79 ± 0.40	5.89 ± 0.33	*p* > 0.05
TG	1.37 ± 0.13	1.52 ± 0.16	*p* > 0.05
TC	4.28 ± 0.19	3.79 ± 0.17	*p* > 0.05
HDL-C	1.35 ± 0.08	1.17 ± 0.06	*p* > 0.05
LDL-C	2.57 ± 0.19	2.32 ± 0.17	*p* > 0.05
K^+^	4.40 ± 0.15	4.23 ± 0.18	*p* > 0.05
Na^+^	139.93 ± 0.49	139.17 ± 0.70	*p* > 0.05
Cl-	97.31 ± 0.73	96.12 ± 0.71	*p* > 0.05
Ca	2.28 ± 0.03	2.27 ± 0.04	*p* > 0.05
P	1.98 ± 0.09	1.97 ± 0.09	*p* > 0.05
hsCRP	4.07 ± 1.09	5.78 ± 1.87	*p* > 0.05
ALT	22 ± 3.73	18.07 ± 3.04	*p* > 0.05
AST	20.07 ± 2.33	18.38 ± 2.46	*p* > 0.05
TP	63.00 ± 1.61	63.48 ± 1.50	*p* > 0.05
ALB	35.42 ± 1.00	35.87 ± 0.85	*p* > 0.05
ALP	75.68 ± 6.98	89.66 ± 10.12	*p* > 0.05

**Figure 3.**
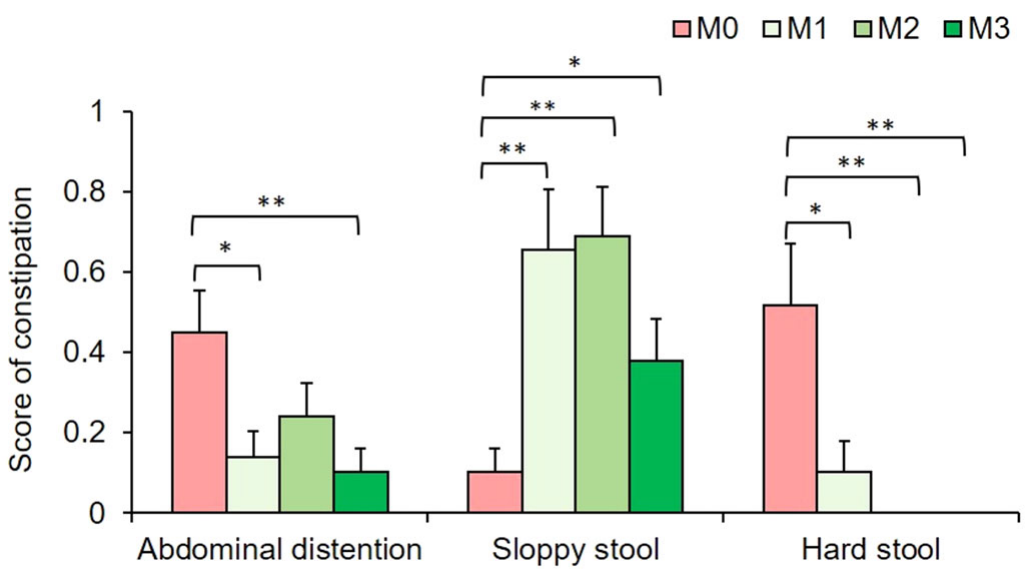
Effects of TXD on stool experience. M0: time of enrollment; M1: end of one month following TXD intervention; M2: end of two months following TXD intervention; M3: end of three months following TXD intervention. *p < 0.05, **p < 0.01.

Next, the patient-recorded stool scores were analyzed at each visit. The results showed that, compared to that of PD patients, TXD greatly improved constipation in PD patients from one month of intervention, decreasing the occurrence of abdominal distention and hard stool, and increasing the occurrence of sloppy stool (Figure 3). At the end of the 3-month intervention, TXD decreased by 80% abdominal distention (*p* < 0.01), increased 2.6-fold sloppy stools (*p* < 0.05) and eliminated hard stool completely (*p* < 0.01, seen in Figure 3).

### Effects of TXD on gut microbiota in PD patients

**Figure 4.**
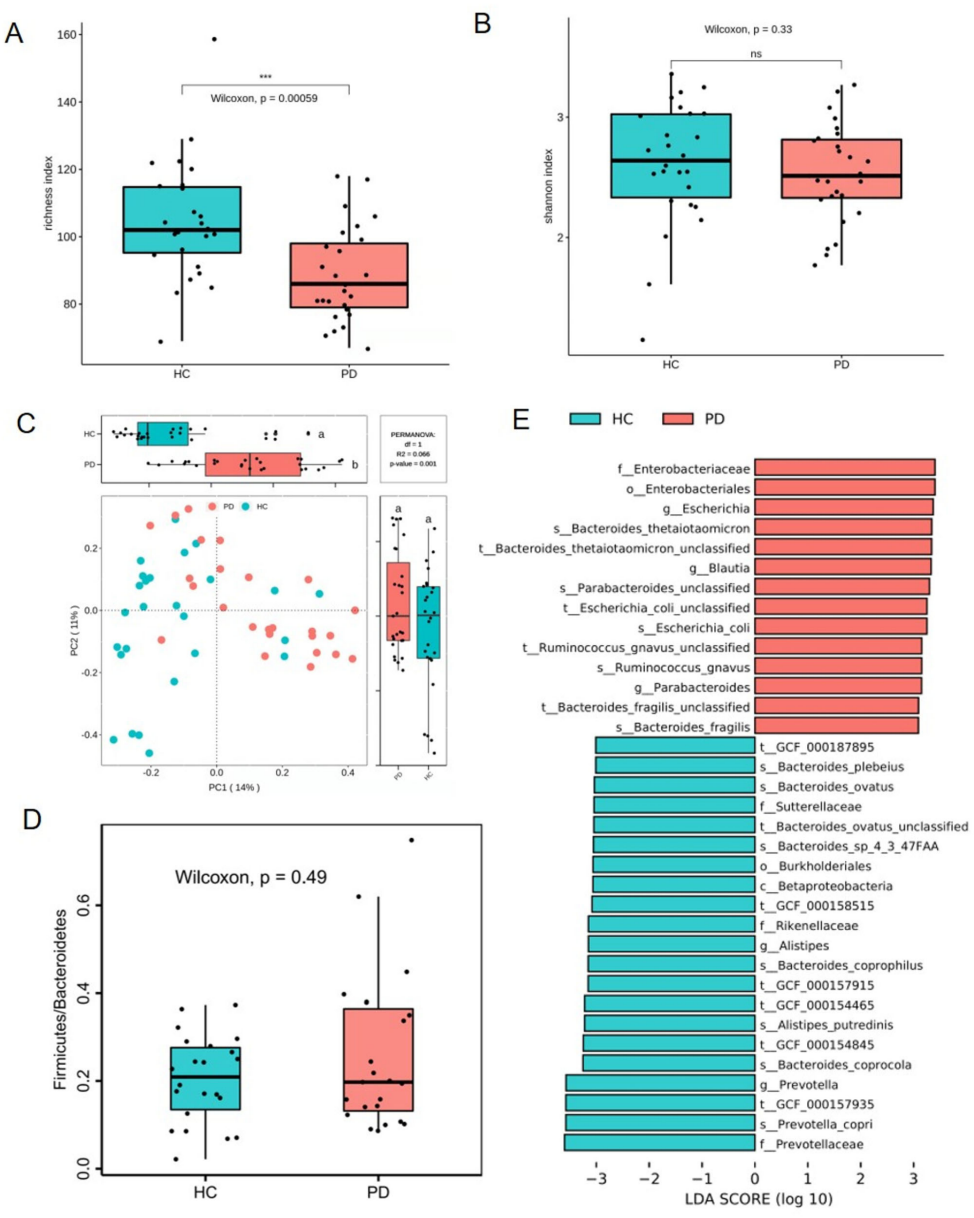
Gut dysbiosis developed in patients with peritoneal dialysis. (A) richness index of gut microbiota. (B) Shannon index of gut microbiota. (C) plots of unweighted unifac-based PCoA. (D) ratio of Firmicutes and Bacteroidetes. (E) LEfSe plot showing specific bacteria. HC: healthy group; PD: peritoneal dialysis patient group. *** *p* < 0.001; ns: non-significance.

Before estimating the effects of TXD on gut microbiota, the changes in microbial structure were determined between PD patients and healthy individuals. Compared with HC group, the richness index, not the Shannon index, showed a significant decrease in PD group’s gut microbiota (Figure 4(A,B)). PCoA assessment showed distinct clustering of intestinal microbe communities for both groups (Figure 4(C)). The ratio of Firmicutes to Bacteroidetes exhibited no difference in PD patients compared to that in healthy individuals (Figure 4(D)). In addition, compared with HC group, the species *Bacteroides thetaiotaomicron*, *Bacteroides fragilis*, *Escherichia coli*, *Parabacteroides unclassified,* and *Ruminococcus gnavus* were greatly enriched in the PD group (Figure 4(E)). According to metagenomic analysis, the biochemical metabolic functions of intestinal flora were predicted using sequencing results. The most statistically significant metabolic pathways are listed in Table 2. Among them, some lead to the disordered metabolism of creatinine and arginine, as well as excessive toxin synthesis (PWY-1269, PWY-5656, CRNFORCAT-PWY, PWY-4981), which further affect renal function through the gut-kidney axis. Together, these results demonstrate that gut dysbiosis developed in PD patients.

**Table 2.** LEfSe plot showing predicted metagenome pathways in PD patients.

Name	*p* Value
ALLANTOINDEG-PWY: superpathway of allantoin degradation in yeast	0.00
CENTFERM-PWY: pyruvate fermentation to butanoate	0.00
CRNFORCAT-PWY: creatinine degradation I	0.00
GLYCOGENSYNTH-PWY: glycogen biosynthesis I (from ADP-D-Glucose)	0.00
HISDEG-PWY: L-histidine degradation I	0.01
HSERMETANA-PWY: L-methionine biosynthesis III	0.00
P124-PWY: Bifidobacterium shunt	0.01
P185-PWY: formaldehyde assimilation III (dihydroxyacetone cycle)	0.01
P23-PWY: reductive TCA cycle I	0.01
P562-PWY: myo-inositol degradation I	0.00
POLYAMINSYN3-PWY: superpathway of polyamine biosynthesis II	0.00
PWY-1269: CMP-3-deoxy-D-manno-octulosonate biosynthesis I KDO	0.01
PWY-1861: formaldehyde assimilation II (RuMP Cycle)	0.01
PWY-241: C4 photosynthetic carbon assimilation cycle, NADP-ME type	0.01
PWY-4981: L-proline biosynthesis II (from arginine)	0.00
PWY-5004: superpathway of L-citrulline metabolism	0.00
PWY-5100: pyruvate fermentation to acetate and lactate II	0.01
PWY-5104: L-isoleucine biosynthesis IV	0.00
PWY-5367: petroselinate biosynthesis	0.00
PWY-5464: superpathway of cytosolic glycolysis (plants), pyruvate dehydrogenase and TCA cycle	0.01
PWY-5656: mannosylglycerate biosynthesis I	0.00
PWY-5676: acetyl-CoA fermentation to butanoate II	0.00
PWY-5692: allantoin degradation to glyoxylate II	0.00
PWY-7209: superpathway of pyrimidine ribonucleosides degradation	0.00
PWY490-3: nitrate reduction VI (assimilatory)	0.00
PWY4LZ-257: superpathway of fermentation (*Chlamydomonas reinhardtii*)	0.00
PWY66-367: ketogenesis	0.00
PWY66-400: glycolysis VI (metazoan)	0.01
PWY66-409: superpathway of purine nucleotide salvage	0.00

**Figure 5.**
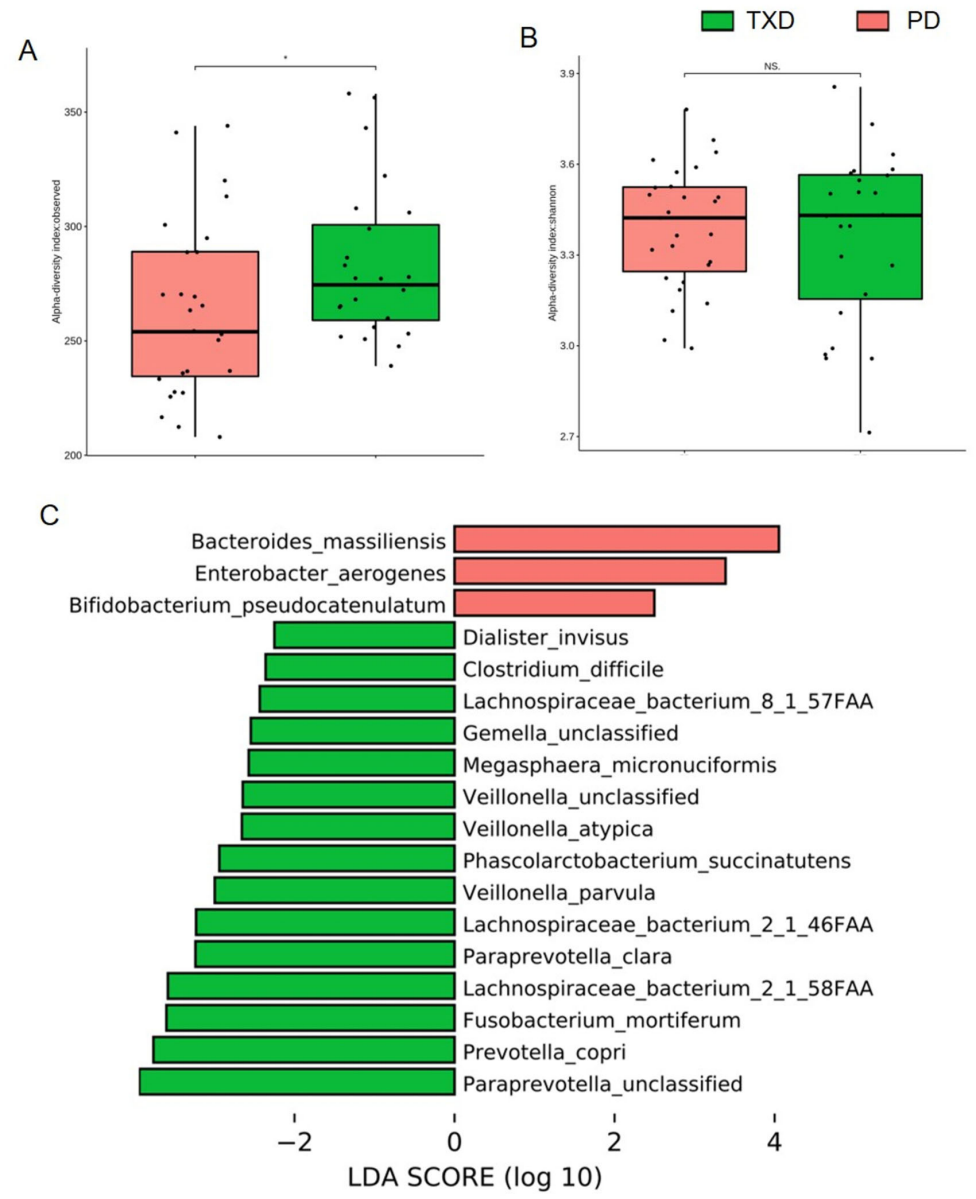
Effects of TXD on gut dysbiosis in PD patients. (A) richness index of gut microbiota. (B) Shannon index of gut microbiota. (C) LEfSe plot showing specific bacteria. TXD: Tiaopi Xiezhuo decoction; PD: peritoneal dialysis patient group; TXD: the group treated by TXD. * *p* < 0.05; NS: non-significance.

Compared with those prior to TXD intervention, the richness index of intestinal flora was significantly increased in PD patients receiving 3-month TXD (*p* = 0.047, Figure 5(A)). However, the Shannon index showed no difference in intestinal flora before and after intervention (*p* = 0.900, Figure 5(B)). To further estimate the change in composition, LEfSe analysis was performed (Figure 5(C)). The enriched intestinal bacteria in the PD group included *Bacteroides massiliensis* and *Enterobacter aerogenes*. Following TXD intervention, the bacteria that accumulated were *Prevotella copri*, *Fusobacterium mortiferum*, *Lachnospiraceae bacterium* 2-1-46FFA*, Lachnospiraceae bacterium* 2-1-58FFA, *Veillonella parvula*, and *Paraprevotella clara*. Consistently, the most enriched metabolic pathways were those related to glucolipid metabolism (Table 3), but not disordered metabolism of creatinine/arginine and excessive toxin synthesis any more (Table 2). These data suggest that gut dysbiosis was greatly modulated by TXD intervention in patients with PD.

**Table 3.** LEfSe plot showing predicted metagenome pathways after the TXD intervention.

Name	*p* Value
ALLANTOINDEG-PWY: superpathway of allantoin degradation in yeast	0.001
P221-PWY: octane oxidation	0.003
PWY-5910: superpathway of geranylgeranyldiphosphate biosynthesis I (*via* mevalonate)	0.015
PWY-621: sucrose degradation III (sucrose invertase)	0.006
PWY-6318: L-phenylalanine degradation IV (mammalian, *via* side chain)	0.004
PWY-6527: stachyose degradation	0.011
PWY-922: mevalonate pathway I	0.039
PWY66-422: D-galactose degradation V (Leloir pathway)	0.041

### Correlation of microbial abundance with clinical characteristics and constipation

**Figure 6.**
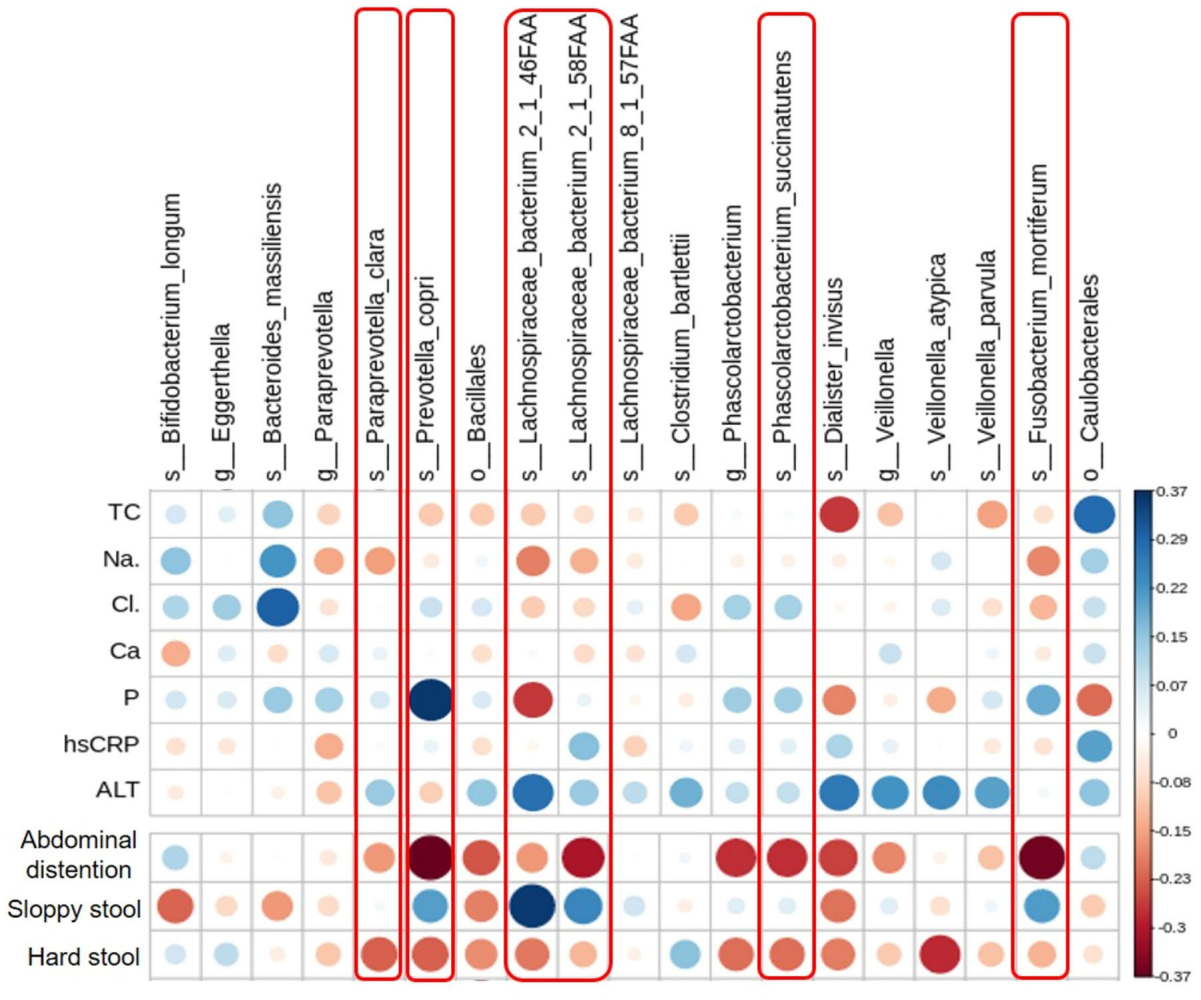
Correlation of TXD-modulated microbial abundance with clinical characteristics and constipation in PD patients. Red frames mark TXD-enriched species with higher correlation with stool conditions. ALT: alanine aminotransferase; Ca: calcium; Cl: chloride; hsCRP: hypersensitive c reactive protein; Na: sodium; P: phosphate; PD: peritoneal dialysis; TC: total cholesterol; TXD: Tiaopi Xiezhuo decoction.

To investigate the association of specific bacteria enriched or diminished by TXD intervention with the effects of TXD on PD patients, the biochemical characteristics or stool scores were analyzed *via* Spearman analysis with specific bacteria in Figure 5(C) and the heat map was drawn (Figure 6). In this heat map, blue indicates a positive correlation, while red indicates a negative correlation; the darker the colour, the greater the correlation, and the larger the circle, the higher the relative abundance. The *p*-values of all dots on the graph were less than 0.05, indicating statistical significance. As shown in Figure 6, most of 3-month TXD-enriched species, including *P. copri, F. mortiferum, L. bacterium* 2-1-46FFA, *and L. bacterium* 2-1-58FFA, had *a* negative correlation with abdominal distention and hard stool, but positive correlation with sloppy stool. These suggested that gut bacteria modulated by TXD may play important roles in alleviating constipation in patients with PD. Differently, although the biochemical characteristics presented no difference between PD and TXD groups, we still found some of them were correlated with TXD-enriched species. For example, *Dialister invisus*, *V. parvula, P. copri, F. mortiferum, L. bacterium* 2-1-46FFA, and *L. bacterium* 2-1-58FFA were negatively correlated with total cholesterol, implying the extension of TXD treatment may have beneficial effects on reducing total cholesterol. *L. bacterium* 2-1-46FFA and *D. invisus* were negatively correlated with the serum levels of phosphate but *P. copri* exhibited a positive correlation.

Collectively, our results suggest that TXD may modulate gut dysbiosis to improve constipation in patients with PD.

## Discussion

Recently, the composition and richness of gut microbiota have been investigated in patients with PD (Stadlbauer et al. 2017; Yacoub et al. 2017; Jiang et al. 2021; Luo et al. 2021). In the present study, we consistently found that gut dysbiosis developed in PD patients in the form of accumulated harmful bacteria (*E. coli* and *B. fragilis*) and diminished beneficial bacteria (*Bacteroides* and *Alistipes*); the analysis of the biochemical metabolic functions of intestinal flora showed that their metabolic pathways lead to the disordered metabolism of creatinine and arginine, as well as excessive toxin synthesis (Table 2), which further affected renal function through the gut-kidney axis. *E. coli* is closely related to peritonitis in patients. A study reports that *E. coli* is the main cause of Gram-negative peritonitis (59.2%), which is often associated with a high probability of mortality and technique failure (Feng et al. 2014). In addition, the toxicity of *E. coli* is more serious than previously thought, leading to a worsened prognosis of *E. coli* peritonitis in PD patients (Valdes-Sotomayor et al. 2003). *B. fragilis*, a major disease-causing *Bacteroides* species, is a common bacterium of appendicitis and septicemia, and can also be isolated from urinary tract infection excretions and peritonitis-infected abdominal exudate (Choi et al. 2016; Valguarnera and Wardenburg 2020; Luo et al. 2021). Other *Bacteroides* species have opposing effects and produce short chain fatty acids (SCFAs), which are involved in gut barrier integrity maintenance, glucose and lipid metabolism, and immunity regulations (Koh et al. 2016). Further investigation into the function of gut bacteria will help understand their role in the pathology of PD.

TXD is developed from the historical Sanhuang Xiexin Decoction, and consists of *R. palmatum*, *C. chinensis*, and *Z. officinale* at a ratio of 1:1:1. Based on the HPLC analysis (Figure 2), there are 15 peaks mainly from *R. palmatum* and *C. chinensis*. Due to the HPLC experimental difference (Tao et al. 2009), we didn’t detect peaks from *Z. officinale*. Among 15 peaks, two of the main compounds in TXD solution are epiberberine and berberine. Berberine is a natural pentacyclic isoquinoline alkaloid with multiple pharmacological effects. Abundant evidence suggests that berberine can modulate gut microbiota in animal and clinical studies, which reduces the composition of harmful bacteria (such as *E. coli* and *Enterococci* bacteria), enriches beneficial bacteria including *Lactobacilli* and *Bifidobacteria*, and elevates SCFA production in the colon (Habtemariam 2020). There is no study to support whether epiberberine has similar effects on the modulation of gut microbiota to berberine. In the present study, our results demonstrated that TXD intervention modulates the structure of gut dysbiosis in patients with PD (Figure 5). Although not consistent with that accumulated by berberine in the references, most of the enriched species by TXD are considered as SCFA-producing bacteria and to promote human health (Thompson et al. 1992; Morotomi et al. 2009; Scheiman et al. 2019; Vacca et al. 2020). This difference may be attributed to other compounds in TXD which may have effects to regulate the composition of gut microbiota.

Among the species enriched by TXD, *Prevotella* is a common spore-free Gram-negative anaerobic bacterium. It is widely found in the oral cavity, intestinal tract, female genital tract, and other parts of the healthy human body, and constitutes the normal flora in these areas (Ley 2016). As members of the Bacteroidetes, *Prevotella* is more common in populations that consume a plant-rich diet, suggesting that it is a beneficial microbe (Wu GD et al. 2011; Martinez et al. 2015). Indeed, *Prevotella* can use xylan, xylose, and carboxymethylcellulose to produce high levels of short-chain fatty acids (Flint et al. 2008). In addition, live *P. copri* was found to improve glucose tolerance in a mouse model (Kovatcheva-Datchary et al. 2015). However, *Prevotella* has also been reported to be associated with immune diseases, especially rheumatoid arthritis, female genital tract inflammation, and oral infection (Kim and Kim 2016). Our data showed that *P. copri* was enriched by TXD and positively correlated with the improvement of constipation in PD patients, but we also found it was positively correlated with the serum levels of phosphate (Figure 6), which are unexpected in patients with chronic kidney disease (CKD). Further studies are needed to understand the role of *Prevotella* in CKD.

Our data demonstrated that the 3-month intervention of TXD decreased the abundance of *E. coli* and enriched multiple beneficial species in patients with PD. It is important to note that intestinal dysbiosis, especially *E. coli*, is associated with an increased risk of peritonitis (Kosmadakis et al. 2018). However, in the present study, because of time limitations, we did not explore whether TXD intervention can reduce the incidence of peritonitis. Additionally, compounds of three herbs in TXD have been reported to reduce proteinuria, inhibit renal fibrosis and improve renal function in previous studies (Li et al. 1997; Wu JS et al. 2014; Ren et al. 2018; Lu et al. 2020; Xiao et al. 2021). Although no significant improvements were detected after 3-month TXD treatment, we still found some TXD-enriched species correlated with part of the biochemical characteristics, for example, total cholesterol. Thus, extending observations to determine the effects of clinical TXD treatment may be necessary for the future.

Clinical data have shown that constipation is one of the most common gastrointestinal disorders among patients with CKD (Kosmadakis et al. 2019). It has been increasingly recognized that chronic constipation in patients with CKD, particularly those receiving PD, affects the mechanical properties of dialysis techniques and predisposes to bacterial intestinal translocation and eventual enteric peritonitis as well as limitations in PD modality (Cano et al. 2007; Kosmadakis et al. 2018, 2019). Recently, increasing studies have suggested that gut microbiota may play important roles in the pathophysiology of constipation, but no clear consensus exists (Ohkusa et al. 2019). The culturing analysis of faecal samples from children with constipation revealed a significantly higher level of *Clostridium* and *Bifidobacterium* species (Zoppi et al. 1998). However, reduced levels of *Bifidobacterium*, *Lactobacillus*, *Bacteroides*, and *Clostridium* species, and increased levels of Enterobacteriaceae were observed in adult constipated patients (Khalif et al. 2005). The results of 16S rRNA gene sequencing demonstrated that constipated patients had a significantly lower level of Bacteroidetes, in particular, *Prevotella*, and an increased level of several species of Firmicutes, including *Lactobacillus* (Zhu et al. 2014). The role of specific intestinal bacteria in the development or improvement of constipation is still poorly understood. The potential mechanism may rely on the products of bacterial fermentation, such as SCFAs. One study in rats has shown that propionate, butyrate, and valerate induce concentration-dependent phasic contractions in the middle and distal colon (Yajima 1985). The effect of SCFAs may be mediated by stimulating the mucosal receptors and acting directly on the colonic smooth muscle (Rondeau et al. 2003). The data in the present study revealed that most of the bacteria enriched by TXD positively correlated with the treatment of constipation, including *P. copri*, *F. mortiferum,* Lachnospiraceae*, V. parvula, and P. clara.* Most of these bacteria are reported to produce SCFA. Whether the beneficial effect of TXD on constipation is associated with SCFA levels in the colon requires further exploration in patients with PD.

## Conclusions

This study is an exploratory work to investigate the beneficial effects of TXD on patients with PD. Our data suggest that TXD treatment may improve constipation by modulating gut microbiota. These findings provide data to support the further application of TXD in the adjuvant treatment of PD.
